# Increase of power conversion efficiency in dye-sensitized solar cells through ferroelectric substrate induced charge transport enhancement

**DOI:** 10.1038/s41598-018-35764-y

**Published:** 2018-11-26

**Authors:** Xiaoyan Liu, Qifeng Zhang, Jiangyu Li, Nagarajan Valanoor, Xiao Tang, Guozhong Cao

**Affiliations:** 1 College of Metallurgy and Materials Engineering, Chongqing Key Laboratory of Nano/Micro Composites and Devices, Chongqing University of Science and Technology, Chongqing, 401331 China; 2 Shenzhen Key Laboratory of Nanobiomechanics, Shenzhen Institutes of Advanced Technology, China Academy of Sciences, Shenzhen, 518055 Guangdong China; 3 Department of Electrical and Computer Engineering, North Dakota State University, Fargo, ND 58108 USA; 4 Department of Mechanical Engineering, University of Washington, Seattle, WA 98195 USA; 5 School of Materials Science and Engineering, University of New South Wales, Sydney, NSW 2052 Australia; 6 Department of Materials Science and Engineering, University of Washington, Seattle, WA 98195 USA

## Abstract

Ferroelectric functionalized dye-sensitized solar cells were fabricated by using a positively-poled LiNbO_3_ substrate coated with ITO (ITO-LiNbO_3_) as a collector electrode and demonstrated enhanced power conversion efficiency. Surface potential properties of TiO_2_ nanoparticle film coated on the ITO-LiNbO_3_ (TiO_2_/ITO-LiNbO_3_) examined by Kelvin probe force microscopy (KPFM) confirmed that a large electric field (a few 10 V/µm) generated from LiNbO_3_ can penetrate through the ITO layer and is applied to TiO_2_ film. This polarization-induced electric field leads to an increased photocurrent density by attracting and promoting electrons to direct transport through the mesoporous TiO_2_ network toward the collector electrode and a decreased charge recombination by facilitating electrons to pass through fewer boundaries of nanoparticles, resulting in high power conversion efficiency. The power conversion efficiency was enhanced by more than 40% in comparison with that without polarization-induced electric field. Incorporating functional ferroelectrics into photovoltaic cells would be a good strategy in improving photovoltaic performance and is applicable to other types of photovoltaic devices, such as perovskite solar cells.

## Introduction

The utilization of solar energy becomes increasingly important, as the fossil and mineral sources are not only limited but also the main sources of environmental pollution. Dye-sensitized solar cells (DSCs) are among the most promising low cost photovoltaic devices to substitute silicon solar cells, the latter is the dominant technology used for commercial solar panels at present^1^. The practical DSC contains broadly a mechanical support coated with transparent conductive oxides (TCOs) as a collector electrode; semiconductor film, usually TiO_2_; a sensitizer absorbed onto the surface of the semiconductor; an electrolyte containing a redox mediator; and a counter electrode capable of regenerating the redox mediator. A nanostructured TiO_2_ film with a high surface area can benefit efficient dye loading and create pathways for electron transport^2,3^. A high photocurrent density as much as 20 mA/cm^2^ can be generated as a result of good light harvesting and electron injection^4,5^. On the other hand, the nanostructured TiO_2_ film with high surface area can also promote charge recombination by decreasing electron diffusion length and hindering charge transport due to the highly random surfaces and boundaries.

Different with the other organic photovoltaic devices, in the DSC the charge generation is done at the TiO_2_-dye interface and the charge transport is completed by the TiO_2_ and the electrolyte. Sun light is absorbed by a dye monolayer located at the junction between the nanostructured TiO_2_ and the triiodide/iodide (I_3_^−^/I^−^) redox electrolyte, leading to an excited sensitizer which injects electrons into the conduction band of the TiO_2_. The processes contributing to the photocurrent are the migration of electrons in the TiO_2_ film toward the collector electrode, the regeneration of oxidized sensitizers by redox couples in electrolyte, and the regeneration of the redox couples by a reduction occurring at the counter electrode. However, the injected electrons may also recombine either with the oxidized sensitizer or with the oxidized redox couple at the TiO_2_ surface, resulting in a loss of cell efficiency. Furthermore, in the DSC the charge transport is mainly forced by electron diffusion because there is no significant electric field existing in the system^6^. Due to trap and recombination limited transport, the electron diffusion through the nanostructured TiO_2_ film is much slower than that in the single crystal TiO_2_ film^7,8^. Extensive studies have shown that the rate of recombination depends on both the electron concentration and the film structure, which can be rationalized in terms of an exponential trap distribution model^9–11^. Enormous progresses have been made through design and synthesis of new dyes^12^, semiconductors^13^ and redox couples^14^, and optimization of semiconductor morphologies^3,15^, leading to a great advancement in power conversion efficiency (PCE). However, there is still a big gap between the practically achieved PCE and the theoretical maximum achievable one (~30%)^16^. Improving the charge transport has become one of the most important issues for approaching a higher efficiency of DSCs.

Recent years, ferroelectric materials have attracted extensive attention for solar cells owing to their unique surface/interface charge properties attributed to spontaneous polarization. The integration of ferroelectrics was initially done with polymer solar cells (PSCs). Yuan *et al*.^17^ and Nalwa *et al*.^18^ obtained an enhanced PCE by introducing a ferroelectric polyvinylidene fluoride (PVDF) layer into PSCs, the former demonstrated an increased open-circuit voltage (*V*_oc_) resulting from an internal electric field ensured by the ferroelectric polymer layer and the later verified an enhanced photocurrent density (*J*_sc_) and fill factor (*FF*) owing to enhanced exciton dissociation by the local electric field of ferroelectric dipoles. Lan *et al*.^19^ observed an increased PCE from inverted PSCs by adopting a dual phase SrTiO_3_/ZnO nanocomposite films as a cathodic buffer layer and claimed that spontaneous polarization of SrTiO_3_ induce a self-built electric field to impede charge recombination at the interface of the active layer and buffer layer. By applying BaTiO_3_/TiO_2_ nanocomposite films as photoanodes, our recent work^2^ demonstrated enhanced PCEs in the DSCs. We concluded that performance improvement in BaTiO_3_ nanocrystalline incorporated cells was due to the increase of electron mobility and decrease of charge recombination caused by the local electric field of ferroelectric BaTiO_3_ dipoles. Moreover, in perovskite solar cells, ferroelectric polarization was demonstrated to improve charge separation inside the absorber and to promote charge transport from CH_3_NH_3_PbI_3_ to TiO_2_^20^, resulting in enhanced photovoltaic performance.

**Figure 1:**
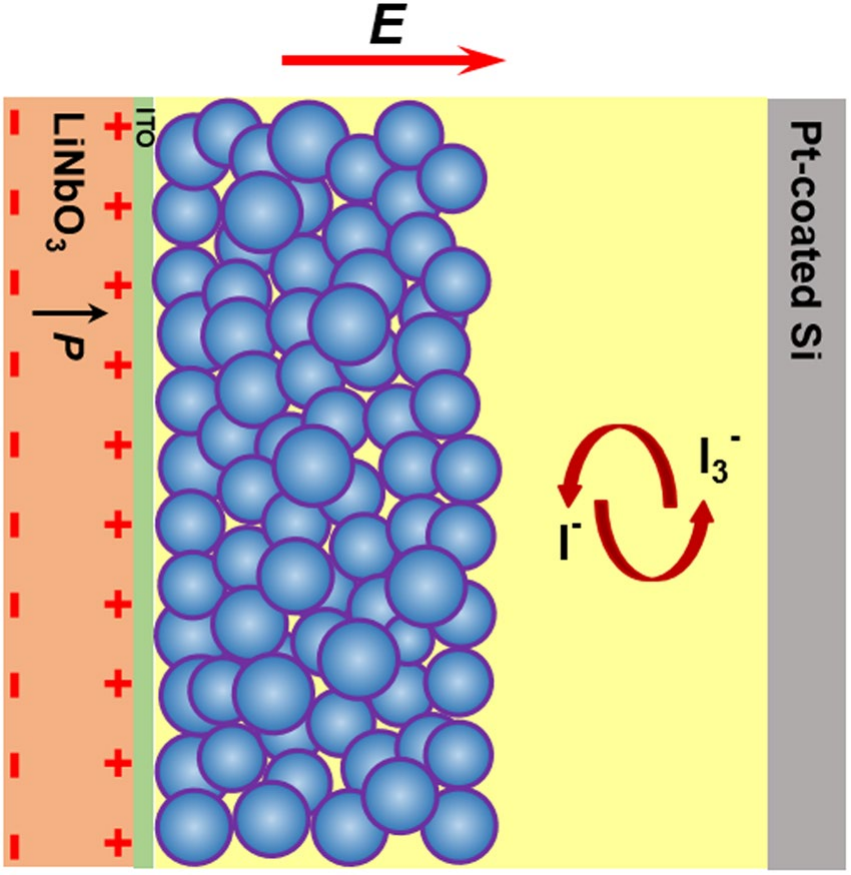
Schematic of DSCs in which ITO coated ferroelectric LiNbO_3_ single crystal with positively-poled surface is used as a collector electrode.

In this paper, we propose a design of ferroelectric functionalized DSCs using a positively-poled (+Z) LiNbO_3_ substrate coated with ITO (ITO-LiNbO_3_) as a collector electrode. Figure 1 illustrates the schematic of the proposed device. After poling, LiNbO_3_ single crystal exhibits a large spontaneous polarization (*P* ~ 75 µC/cm^2^) along the crystallographic Z-axis^21^ which generates high density bound charges at the surface^22,23^. When the poled surface of LiNbO_3_ single crystal is deposited with an ITO layer followed by the coating of a TiO_2_ nanoparticle film, the bound charges at the poled surface are partially or completely compensated by the ITO, and the screening charge sheet generates an electric field penetrating into the nanostructured TiO_2_ film. It is worth mentioning that, at the interface of ITO-LiNbO_3_ and nanostructured TiO_2_ film, the polarization-induced charges will not be fully compensated by the low-concentration free charges in semiconductor, which leads to the formation of an uncompensated internal field in the TiO_2_ nanoparticles. Such a polarization-induced electric field is anticipated to favor charge transport in DSCs. LiNbO_3_ single crystal is employed for this study because of its advantages in material characteristics such as (1) the possession of a large spontaneous polarization (*P*) of 75 µC/cm^2^ arising from the ionic displacements within the crystal lattice, which is highly stable due to a large coercive field (21 kV/mm, the electric field for polarization reversal of congruent LiNbO_3_), resulting in a permanent polarization without change of orientation and magnitude, and (2) its high Curie temperature (1140 °C, the phase transition point from paraelectrics to ferroelectrics) which enables to maintain ferroelectric properties through a sintering process at the temperature ranging of 350–500 °C to form a mesoporous TiO_2_ network that provides pathways for charge transport. In addition, non-doped congruent LiNbO_3_ single crystal shows a good optical transmittance in the visible region (Figure S1). In our work, by using the ITO-LiNbO_3_ (+Z) as a collector electrode, the PCE of DSCs increases by 42.9% resulted from both increased photocurrent density (*J*_sc_) and fill factor (*FF*). The possible mechanism and influences of the polarization-induced electric field on the PCE have been discussed.

## Results and Discussion

### Polarization-induced electric field

Ferroelectric materials have spontaneous polarization without the need of an external electric field. The spontaneous polarization charges can be presented as ${\sigma }_{pol}=P\,n$, where *P* is the polarization vector and n is the unit normal to the surface. When a ferroelectric surface contacts with a conductive layer (i.e., ITO), polarization charges at the ferroelectric surface will be screened by free charges in the conductive layer. The distribution of screening charges in the conductive layer, very near the interface with the ferroelectric, leads to several important thickness-dependent electrical phenomena in ferroelectrics. A depolarization field can be formed due to the slight offset between the polarization charges at the ferroelectric surface and the screening charges in the conductive layer^24–26^. Assume that the ferroelectric is homogeneously-poled, the interface states and space charge in the ferroelectric may be ignored^25^, so that the polarization charges are localized at the ferroelectric/conductive-layer interface. The depolarization field (*E*_*d*_) in the ferroelectric can be represented as

1 $${E}_{d}=-\,\frac{p}{{\varepsilon }_{f}}(\frac{2{\varepsilon }_{f}/d}{2{\varepsilon }_{f}/d+{\varepsilon }_{c}/{l}_{s}})\,$$

where *d* is the thickness of ferroelectric plate, *l*_*s*_ is the screening length (space charge extent in conductive layer), and *ε*_*f*_ and *ε*_*c*_ are the relative dielectric constants of the ferroelectric and the conductor, respectively^25^. It is clear that the magnitude of the depolarization field is strongly plate thickness dependent. In our work, we used 0.3-mm-thick LiNbO_3_ (i.e., *d* = 0.3 mm) and 50 nm-thick ITO (which gives rise to *l*_*s*_ = 0.05 nm)^27^, i.e., $d\gg {l}_{s}$ and thus the depolarization field is predicted to disappear. In this case, screening charges reside at the ferroelectric/conductive-layer interface, completely compensating for the spontaneous polarization *P* in the ferroelectric. The screening charges (${\sigma }_{s},|{\sigma }_{s}|=|{\sigma }_{pol}|$), generates an electric field (*E*) within the adjacent TiO_2_ layer, which is related to the polarization-induced electric field on the ferroelectric surface.

**Figure 2:**
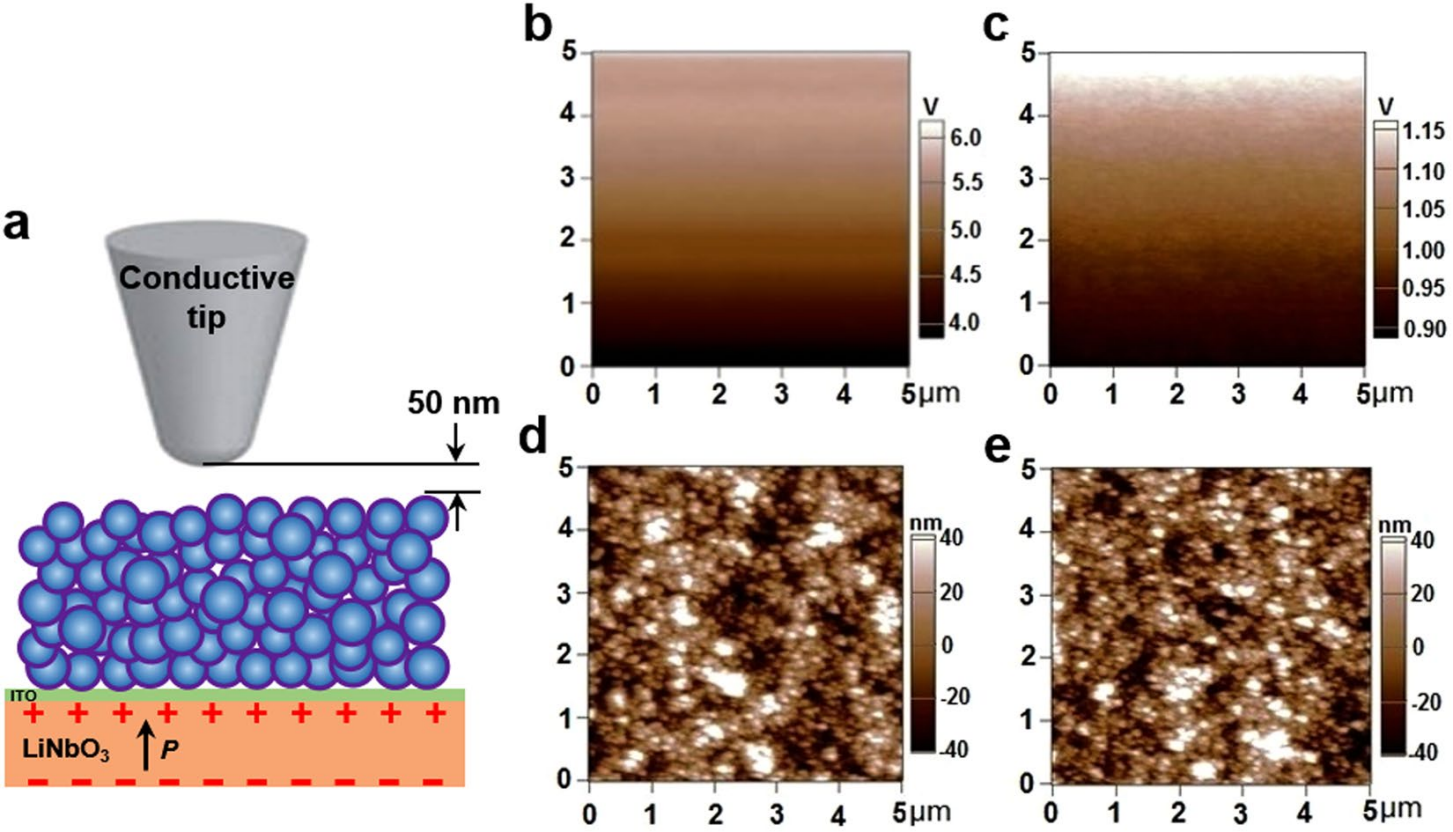
(**a**) Schematic of the Kelvin probe force microscopy (KPFM) measurement. Surface potential images of TiO_2_/ITO-LiNbO_3_ (**b**) and TiO_2_/ITO-glass (**c**). Topographic images of TiO_2_/ITO-LiNbO_3_ (**d**) and TiO_2_/ITO-glass (**e**). The images (**b**–**e**) were taken after subjected to the sintering process.

To verify the electric field induced by the spontaneous polarization of LiNbO_3_ and whether it can penetrate through the ITO layer and TiO_2_ film, surface potential properties of the photoanode after subjected to the sintering process were investigated with Kelvin probe force microscopy (KPFM). As shown in Fig. 2, surface potential of the TiO_2_/ITO-LiNbO_3_ (+Z, Fig. 2b) was much larger than that of the TiO_2_/ITO-glass (Fig. 2c), indicating a potential enhancement on the TiO_2_ film due to the employment of LiNbO_3_. Topographic images (Fig. 2d and e) display a very similar morphology of TiO_2_ nanoparticles for these two samples, suggesting that topography effect on the surface potential is negligible. The electric field generated from the LiNbO_3_ at the tip scanning level can be estimated to be a few 10 V/µm. Variations of surface potential over the scan area were observed from both samples, which might be caused by an AC voltage generally applied to the tip while scanning. KPFM though is not for a quantitative measurement of surface potential^28^, the results clearly confirmed that the electric field originating from the LiNbO_3_ is significantly large and can penetrate through the ITO layer and TiO_2_ film. In other words, the TiO_2_ film is “immersed” in an electric field in view of the spontaneous polarization of LiNbO_3_.

To demonstrate that the polarization-induced electric field can be introduced into DSCs for charge transport promotion, two types of cells were fabricated and studied in this work: ferro-based cell has a configuration of TiO_2_/ITO-LiNbO_3_ (+Z), while glass-based cell is composed of TiO_2_/ITO-glass. For each type, more than five cells were adopted for characteristics of charge transport properties and photovoltaic performance.

### Optical transmittance of collector electrodes

**Figure 3:**
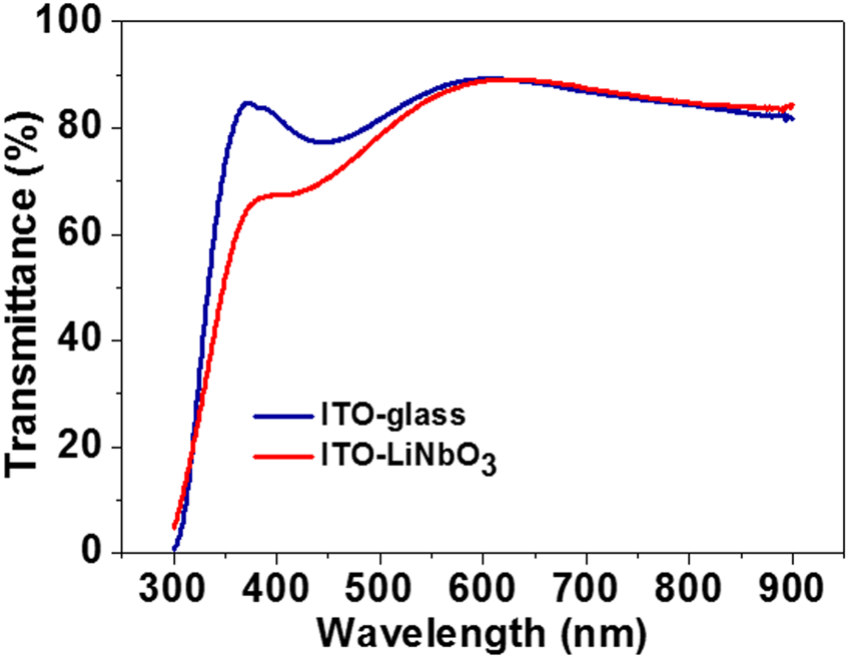
The UV-vis spectra of the collector electrodes of ITO-LiNbO_3_ and ITO-glass.

Optical properties of ITO-LiNbO_3_ and ITO-glass were measured and compared, and their UV-vis transmittance spectra were shown in Fig. 3. It was found that both electrodes have good optical transmittance in the effective absorption wavelength region of N719, although the collector electrode of ITO-LiNbO_3_ shows a lower transmittance at the wavelength range of 320–600 nm.

### Charge transport properties

**Figure 4:**
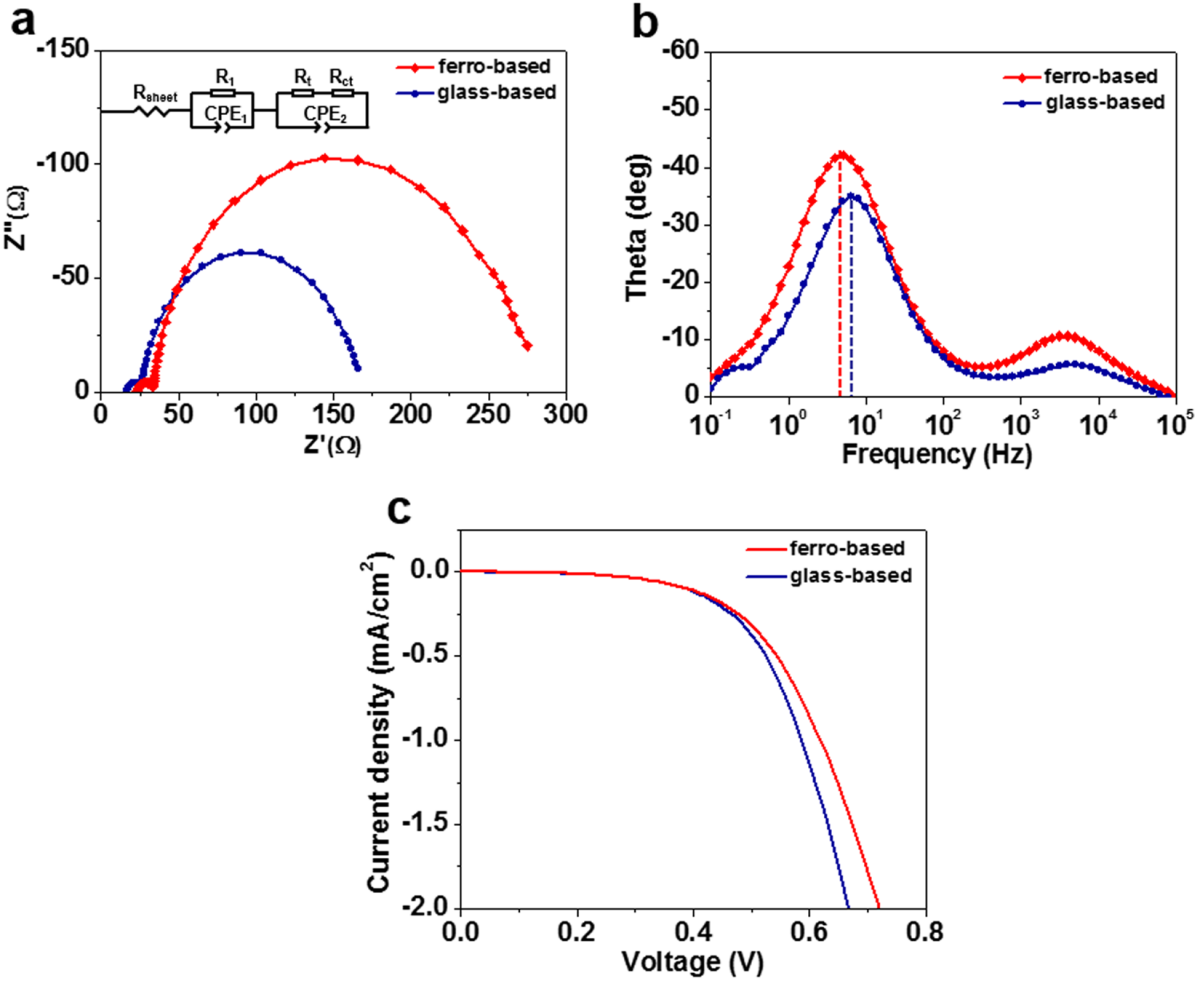
(**a**) Nyquist plot and (**b**) Bode plot of the ferro-based and glass-based cells under open circuit condition (*V*_oc_), and (**c**) photocurrent density-voltage (*J*-*V*) curves of the ferro-based and glass-based cells under dark conditions.

Effects of polarization-induced electric field on the charge transport properties in the DSCs were investigated by electrochemical impedance spectroscopy (EIS). As a well-established technique, the EIS has been widely employed to study the kinetics of electrochemical and photoelectrochemical systems including DSCs^29–32^. Under the EIS measurement with a forward bias in dark, electrons are injected into the conduction band of TiO_2_ nanoparticles from the collector electrode and then transported through the mesoscopic TiO_2_ network. At the same time, some of the injected electrons react with I_3_^−^ in the electrolyte, which is related to the recombination process. The Nyquist plot typically consists of two semicircles, which is fitted by an equivalent circuit inserted in Fig. 4(a). The first small semicircle corresponds to the charge transfer resistance at counter electrode/electrolyte interface (*R*_1_) at a high frequency (>10^3^ Hz). The second large semicircle corresponds to the charge transport resistance within the TiO_2_ film (*R*_t_) at 10^3^-10^2^ Hz and the charge transfer resistance at the TiO_2_/electrolyte interface (*R*_ct_) around 10^1^ Hz. *R*_t_ denotes how effective the electrons in the conduction band of TiO_2_ network move along with the TiO_2_ network, while *R*_ct_ reflects the charge recombination^33^. Under the open circuit condition (*V*_oc_), a relatively high carrier density results in a low diffusion resistance, the *R*_t_ is not usually detected in Nyquist plot due to its small value compared to other two charge transfer resistances *R*_1_ and *R*_ct_^34^. Therefore, the second large semicircle is mainly attributed to the charge transfer resistance *R*_ct._

Figure 4 shows the representative results of EIS measurements at the *V*_oc_. An increase of *R*_ct_ was obtained from the ferro-based cell with the use of LiNbO_3_ as shown in Fig. 4a. The larger the amount of *R*_ct_, the more difficult the recombination of the electrons and holes, which results in decreased charge recombination^35,36^. Figure 4b shows the Bode plots of the cells, indicating a longer lifetime for the ferro-based cell (32 ms) than the glass-based cell (24 ms). The electron lifetime (τ) can be determined by using the curve peak of the spectrum following equation^37^:

2 $${\rm{\tau }}=\frac{1}{2\pi {f}_{peak}}$$

where *f*_peak_ is the peak frequency at the minimum phase angle, 4.915 Hz for the ferro-based cell and 6.478 Hz for the glass-based cell. Both the increases in the *R*_ct_ and τ lead to a reduced interface charge recombination in ferro-based cells.

Figure 4c displays *J*-*V* curves of the cells measured under dark conditions, showing dark currents caused by the reaction of injected electrons with I_3_^−^ in the electrolyte^38^. The dark current was smaller in the ferro-based cell than that in the glass-based cell. This is in keeping with the EIS results, which further demonstrates that charge recombination was impeded by the polarization-induced electric field in ferro-based cells.

### Photovoltaic performance

**Figure 5:**
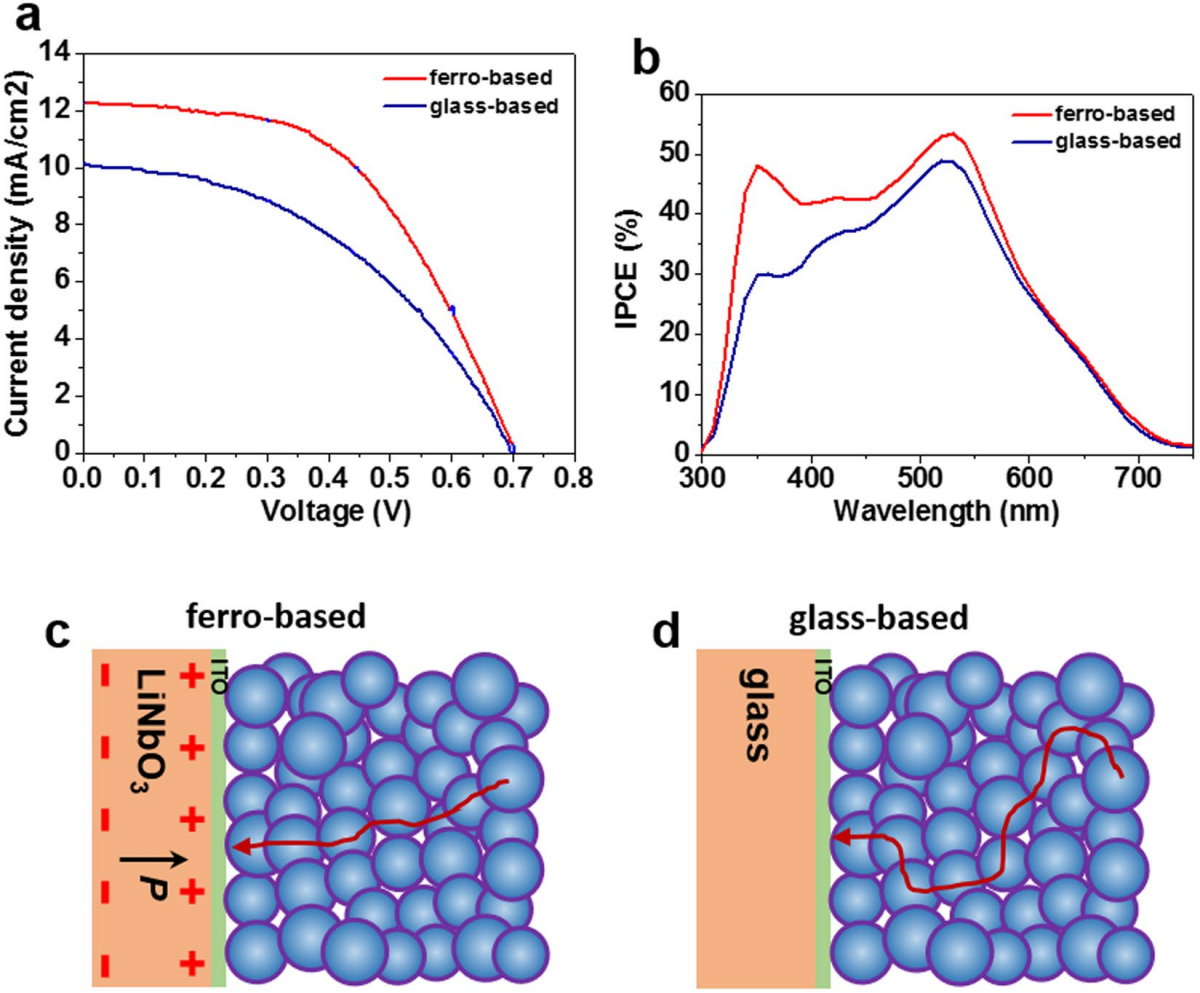
(**a**) Photocurrent density-voltage (*J*-*V*) curves and (**b**) incident photon-to-current conversion efficiency (IPCE) spectra of the ferro-based and glass-based cells under AM 1.5 with 100 mW/cm^2^. Schematics illustrating electron transport through the TiO_2_ network in ferro-based (**c**) and glass-based (**d**) cells.

Figure 5 shows the photocurrent density-voltage (*J*-*V*) characteristics and the corresponding incident monochromatic photon-to-electric current conversion efficiency (IPCE) spectra of the ferro-based and glass-based cells, and their photovoltaic performance parameters are summarized in Table 1. Ferro-based cell had a PCE of 4.46%, which is 42.9% higher than that of 3.12% for glass-based cell. Such an efficiency enhancement in ferro-based cells was mainly attributed to the increased *J*_sc_. It is known that *J*_sc_ is the integration of the IPCE over the absorption range. As expected, ferro-based cells show higher IPCE values comparing with glass-based cells, despite the lower optical transmittance in LiNbO_3_, which is in a same trend to that of *J*_sc_. The increased *J*_sc_ can be ascribed to the existence of a polarization-induced electric field from ferroelectric LiNbO_3_ in the ferro-based cell, in which electrons transport along the TiO_2_ network are enforced by the electric field toward the electron collection electrode and consequently the transport involves less charge recombination at the TiO_2_/electrolyte interfaces (Fig. 5d), resulting in a larger photocurrent density.

**Table 1:** Photovoltaic performance parameters of ferro-based and glass-based cells.

device	*J*_sc_ (mA/cm^2^)	*V*_oc_ (V)	*FF*	η (%)	*R*_s_ (kΩ cm^2^)	*R*_sh_ (kΩ cm^2^)
ferro-based	12.27	0.7	0.52	4.46	0.0210	0.522
glass-based	11.12	0.7	0.44	3.12	0.0278	0.232

In addition to the increase in *J*_sc_, fill factor (*FF*) of ferro-based cells also increases and contributes to the higher PCE. The *FF* reflects electrical and electrochemical losses occurring during operation of the DSC, it can be contributed by series and shunt resistance in the device. The shunt resistance (*R*_sh_) is for the resistance related to the back electron transfer across the TiO_2_/electrolyte interface^39^, and the series resistance (*R*_s_) is mainly attributed to the sheet resistance of TCO substrates. Increasing *R*_sh_ and decreasing *R*_s_ will lead to a higher *FF*, thus resulting in a greater efficiency. The *R*_sh_ and *R*_s_ can be obtained by calculating the inverse of the slopes of *J*-*V* characteristics near *J*_sc_ and *V*_oc_, respectively. Compared with the glass-based cell, the ferro-based cell presents a slightly smaller *R*_s_ but an apparently larger *R*_sh_, resulting in a larger *FF* (Table 1). The larger *R*_sh_ originating from reduced charge recombination can be ascribed to the field-assisted charge collection which leads to less opportunities for the electrons to back react at the TiO_2_/electrolyte interfaces. It should also be noted that the overall PCEs are not high due to the small *FF*. The large *R*_s_ caused by the ITO might be the reason for the small *FF*. To confirm this speculation, resistivity of the ITO and FTO (commonly used as a TCO of collector electrodes in DSCs) before and after the sintering process were measured and compared. The results show that the resistivity of the ITO increased slightly after subjected to the sintering process (2.767 and 2.865 Ω·cm respectively, before and after sintering), however, it is 2.2 times of that of FTO (1.293 Ω·cm after the same sintering process). The comparatively large resistivity of the ITO leads to the small *FF* thus the overall low PCEs from this work.

## Conclusions

In summary, we report ferroelectric functionalized DSCs, in which a positively-poled LiNbO_3_ single crystal coated with ITO was used as a collector electrode, have better performance due to higher photocurrent density and fill factor. The large spontaneous polarization of LiNbO_3_ generates an electric field that can penetrate through the ITO layer and into TiO_2_ film, giving rise to two impacts on electron transport in the mesoporous TiO_2_ network: enhanced photocurrent density by attracting electrons to transport through the mesoporous TiO_2_ network toward the collector electrode and decreased recombination chance by facilitating electrons to pass through fewer boundaries of TiO_2_ nanoparticles. Further work is necessary to optimize the incorporation of ferroelectrics into solar cells in order to take full advantages of polarization-induced electric field to further enhance the PCE of DSCs.

## Materials and Methods

### Preparation of collector electrodes

LiNbO_3_ single crystal of congruent composition (YAMAJYU Ceramics Co., Ltd, Japan) was uniformly poled along with the crystallographic Z-axis and cut perpendicular to the polar axis, and then polished to a plate with the thickness of 0.3 mm. Following the polishing, the plate was diced into 2 × 2 cm pieces and ultrasonic cleaned in acetone, methanol and deionized (DI) water sequentially, and then blown dry with nitrogen. After cleaning, a 50-nm-thick indium tin oxide (ITO) film was sputtered onto the positively-poled (+Z) surface of LiNbO_3_ single crystals. For comparison, ITO-coated glasses were also prepared following the same process.

### Fabrication of solar cells

TiO_2_ paste was prepared based on the literature^40^ using commercial TiO_2_ powder (P25). Doctor-blading method was adopted to make a TiO_2_ film onto the ITO-LiNbO_3_ or ITO-glass. The film was first dried at 150 °C for 30 min and then annealed at 350 °C for 1 h at the heating rate of 10 °C/min to remove the organic additives and to create a mesoporous network of TiO_2_. Owing to the thermal restriction of ITO, 350 °C for 1 hr was applied to anneal the TiO_2_ films in this work. It has been confirmed that, the organic residues can be entirely removed, and anatase TiO_2_ phase structure can be formed after annealing at 350 °C^41^.

The resulting TiO_2_ film was then sensitized with a 0.3 mM solution of N719 dye (Solaronix) for 24 h. The N719 dye was dissolved in ACN and tBA (volume ratio of 1:1) as a standard dye solution. The sensitized film was assembled with a counter electrode made of a Pt-coated silicon after removing excess dye with ethanol and drying with air flow. A 30-μm-thick spacer was used to separate the sensitized film and the counter electrode, and the gap between them was filled with an electrolyte composed of 0.6 M tetrabutylammomium iodide, 0.1 M lithium iodide, 0.1 M iodine and 0.5 M 4-tert-butylpyridine in acetonitrile.

### Characterization

The morphology of the glass, LiNbO_3_, ITO-glass and ITO-LiNbO_3_ (Figure S2) was characterized by atomic force microscopy (AFM, Asylum Research Cypher S) at an ac mode. The surface potential properties of TiO_2_ film coated on collector electrodes after the sintering process were characterized by Kelvin probe force microscopy (KPFM, Asylum Research Cypher S), the corresponding surface morphology of the films can be obtained simultaneously with the KPFM. A conductive cantilever with a spring constant of 2 N/m and tip radius of 10 nm was used. Surface potential properties were characterized by scanning the TiO_2_ films with the conductive tip at a constant level of 50 nm from the film.

The optical transmittance of the LiNbO_3_ (Figure S1), ITO-glass and ITO-LiNbO_3_ was characterized by UV-vis spectrometer. Electrochemical impedance spectroscopy (EIS) was performed using a Solartron 1287 A equipped with a Solartron 1260 FRA/impedance analyzer to investigate charge transport properties in the DSCs. The frequency range was set from 10^−1^ to 10^5^ Hz and the oscillation potential amplitude was 10 mV. The photovoltaic performance of the DSCs was characterized by using a HP 4155 A programmable semiconductor parameter analyzer equipped with an AM 1.5 simulated sunlight with a power density of 100 mW/cm^2^. The incident monochromatic photon-to-electron conversion efficiency (IPCE) plotted as a function of excitation wavelength were recorded on a QTest Station 1000 ADI system (Crowntech, Inc.) equipped with a 300 W Xe lamp. The monochromatic photocurrent-wavelength measurements were carried out by placing a monochromator, assisted by an automatic filter wheel, between the DSCs and the light source. A mask was used to enable an illuminated active area of 0.196 cm^2^.

## Electronic supplementary material

Increase of power conversion efficiency in dye-sensitized solar cells through ferroelectric substrate induced charge transport enhancement

## References

[1] Yue JY (2017). Enhanced photovoltaic performances of the dye-sensitized solar cell by utilizing rare-earth modified tin oxide compact layer. Org Electron.

[2] Feng KY (2017). Ferroelectric BaTiO3 dipole induced charge transfer enhancement in dye-sensitized solar cells. J Power Sources.

[3] Zhang QF, Cao GZ (2011). Nanostructured photoelectrodes for dye-sensitized solar cells. Nano Today.

[4] Meyer GJ (2010). The 2010 Millennium Technology Grand Prize: Dye-Sensitized Solar Cells. Acs Nano.

[5] Nazeeruddin MK (1993). Conversion of Light to Electricity by Cis-X2bis(2,2′-Bipyridyl-4,4′-Dicarboxylate)Ruthenium(Ii) Charge-Transfer Sensitizers (X = Cl-, Br-, I-, Cn-, and Scn-) on Nanocrystalline Tio2 Electrodes. J Am Chem Soc.

[6] Wang M, Chen P, Humphry-Baker R, Zakeeruddin SM, Gratzel M (2009). The Influence of Charge Transport and Recombination on the Performance of Dye-Sensitized Solar Cells. Chemphyschem.

[7] Konenkamp R (2000). Carrier transport in nanoporous TiO2 films. Phys Rev B.

[8] Kopidakis N, Schiff EA, Park NG, van de Lagemaat J, Frank AJ (2000). Ambipolar diffusion of photocarriers in electrolyte-filled, nanoporous TiO2. J Phys Chem B.

[9] Bisquert J, Zaban A, Salvador P (2002). Analysis of the mechanisms of electron recombination in nanoporous TiO2 dye-sensitized solar cells. Nonequilibrium steady-state statistics and interfacial electron transfer via surface states. J Phys Chem B.

[10] Shen Y, Nonomura K, Schlettwein D, Zhao C, Wittstock G (2006). Photoelectrochemical kinetics of eosin Y-sensitized zinc oxide films investigated by scanning electrochemical microscopy. Chem-Eur J.

[11] Bisquert J (2008). Physical electrochemistry of nanostructured devices. Phys Chem Chem Phys.

[12] Hailu YM, Shie WR, Nachimuthu S, Jiang JC (2017). New Insights into Organic Dye Regeneration Mechanism in Dye Sensitized Solar Cells: A Theoretical Study. Acs Sustain Chem Eng.

[13] Vittal R, Ho KC (2017). Zinc oxide based dye-sensitized solar cells: A review. Renew Sust Energ Rev.

[14] Hwang DK, Nam JE, Jo HJ, Sung SJ (2017). Quasi-solid state electrolyte for semi-transparent bifacial dye-sensitized solar cell with over 10% power conversion efficiency. J Power Sources.

[15] Park K, Zhang QF, Xi JT, Cao GZ (2015). Enhanced charge transport properties by strengthened necks between TiO2 aggregates for dye sensitized solar cells. Thin Solid Films.

[16] Snaith HJ (2010). Estimating the Maximum Attainable Efficiency in Dye-Sensitized Solar Cells. Adv Funct Mater.

[17] Yuan YB (2011). Efficiency enhancement in organic solar cells with ferroelectric polymers. Nat Mater.

[18] Nalwa KS (2012). Enhanced charge separation in organic photovoltaic films doped with ferroelectric dipoles. Energ Environ Sci.

[19] Lan JL (2014). The effect of SrTiO3:ZnO as cathodic buffer layer for inverted polymer solar cells. Nano Energy.

[20] Kim HS (2015). Ferroelectric Polarization in CH3NH3PbI3 Perovskite. J Phys Chem Lett.

[21] Gopalan V, Mitchell TE, Furukawa Y, Kitamura K (1998). The role of nonstoichiometry in 180 degrees domain switching of LiNbO3 crystals. Appl Phys Lett.

[22] Liu Xiaoyan, Kitamura Kenji, Terabe Kazuya (2006). Surface potential imaging of nanoscale LiNbO3 domains investigated by electrostatic force microscopy. Applied Physics Letters.

[23] Liu XY, Terabe K, Kitamura K (2006). Surface potential properties on near-stoichiometric LiNbO3 crystals with nanoscale domain-engineered structures. J Electroceram.

[24] Junquera J, Ghosez P (2003). Critical thickness for ferroelectricity in perovskite ultrathin films. Nature.

[25] Mehta RR, Silverman BD, Jacobs JT (1973). Depolarization Fields in Thin Ferroelectric Films. J Appl Phys.

[26] Kornev, I., Fu, H. X. & Bellaiche, L. Ultrathin films of ferroelectric solid solutions under a residual depolarizing field. *Phys Rev Lett* **93** (2004). 10.1103/PhysRevLett.93.196104 15600856

[27] Kim, D. J. *et al*. Polarization relaxation induced by a depolarization field in ultrathin ferroelectric BaTiO3 capacitors. *Phys Rev Lett* **95** (2005). 10.1103/PhysRevLett.95.237602 16384347

[28] Wen Huan Fei, Li Yan Jun, Arima Eiji, Naitoh Yoshitaka, Sugawara Yasuhiro, Xu Rui, Cheng Zhi Hai (2017). Investigation of tunneling current and local contact potential difference on the TiO2(110) surface by AFM/KPFM at 78 K. Nanotechnology.

[29] Bisquert J (2002). Theory of the impedance of electron diffusion and recombination in a thin layer. J Phys Chem B.

[30] Bisquert J, Vikhrenko VS (2004). Interpretation of the time constants measured by kinetic techniques in nanostructured semiconductor electrodes and dye-sensitized solar cells. J Phys Chem B.

[31] Han LY, Koide N, Chiba Y, Mitate T (2004). Modeling of an equivalent circuit for dye-sensitized solar cells. Appl Phys Lett.

[32] Wang Q (2006). Characteristics of high efficiency dye-sensitized solar cells. J Phys Chem B.

[33] Lv Q (2012). Promotion effect of TiO2 on catalytic activity and stability of Pt catalyst for electrooxidation of methanol. J Power Sources.

[34] Hoshikawa T, Yamada M, Kikuchi R, Eguchi K (2005). Impedance analysis of internal resistance affecting the photoelectrochemical performance of dye-sensitized solar cells. J Electrochem Soc.

[35] van de Lagemaat J, Park NG, Frank AJ (2000). Influence of electrical potential distribution, charge transport, and recombination on the photopotential and photocurrent conversion efficiency of dye-sensitized nanocrystalline TiO2 solar cells: A study by electrical impedance and optical modulation techniques. J Phys Chem B.

[36] Kern R, Sastrawan R, Ferber J, Stangl R, Luther J (2002). Modeling and interpretation of electrical impedance spectra of dye solar cells operated under open-circuit conditions. Electrochim Acta.

[37] Bisquert J, Fabregat-Santiago F, Mora-Sero I, Garcia-Belmonte G, Gimenez S (2009). Electron Lifetime in Dye-Sensitized Solar Cells: Theory and Interpretation of Measurements. J Phys Chem C.

[38] Ito, S. *et al*. Control of dark current in photoelectrochemical (TiO2/I–I3-)) and dye-sensitized solar cells. *Chem Commun (Camb)*, 4351–4353, 10.1039/b505718c (2005). 10.1039/b505718c 16113745

[39] Gratzel M (2001). Photoelectrochemical cells. Nature.

[40] Ito S (2007). Fabrication of screen-printing pastes from TiO2 powders for dye-sensitised solar cells. Prog Photovoltaics.

[41] Xi J, Dahoudi NA, Zhang Q, Sun Y, Cao G (2012). Effect of annealing temperature on the performances and electrochemical properties of TiO2 dye-sensitized solar cells. Science of Advanced Materials.

