# Supplementary material for: Increase of power conversion efficiency in dye-sensitized solar cells through ferroelectric substrate induced charge transport enhancement
Source: Sci Rep. 2018 Nov 26;8:17389. doi: 10.1038/s41598-018-35764-y (PMC6255840; doi:10.1038/s41598-018-35764-y)
Supplement: Supplementary file 1 — Increase of power conversion efficiency in dye-sensitized solar cells through ferroelectric substrate induced charge transport enhancement [file 41598_2018_35764_MOESM1_ESM.docx]

**Supplementary Information**

**Increase of power conversion efficiency in dye-sensitized solar cells through ferroelectric substrate induced charge transport enhancement**

Xiaoyan Liu^a,b*^, Qifeng Zhang^c^, Jiangyu Li^d,b^, Nagarajan Valanoor^e^, Xiao Tang^a^,

Guozhong Cao^f,*^

^a^College of Metallurgy and Materials Engineering, Chongqing Key Laboratory of Nano/Micro

Composites and Devices, Chongqing University of Science and Technology, Chongqing 401331, China

^b^Shenzhen Key Laboratory of Nanobiomechanics, Shenzhen Institutes of Advanced Technology, China

Academy of Sciences, Shenzhen 518055, Guangdong, China

^c^Department of Electrical and Computer Engineering, North Dakota State University, Fargo, ND 58108, USA

^d^Department of Mechanical Engineering, University of Washington, Seattle, WA 98195, USA

^e^[School of Materials Science and Engineering](https://www.researchgate.net/institution/UNSW_Sydney/department/School_of_Materials_Science_and_Engineering), University of New South Wales, NSW 2052, Australia

^f^Department of Materials Science and Engineering, University of Washington, Seattle, WA 98195, USA

^*^Corresponding authors: Xiaoyan Liu and Guozhong Cao

E-mail address: [xyliu@cqust.edu.cn](mailto:xyliu@cqust.edu.cn) and [gzcao@u.washington.edu](mailto:gzcao@u.washington.edu)

Figure S1 The UV-vis spectra of LiNbO_3_ single crystal. The LiNbO_3_ single crystal shows a good optical transmittance in the visible region confirmed that it can be used as a substrate of collector electrodes in DSCs.

**
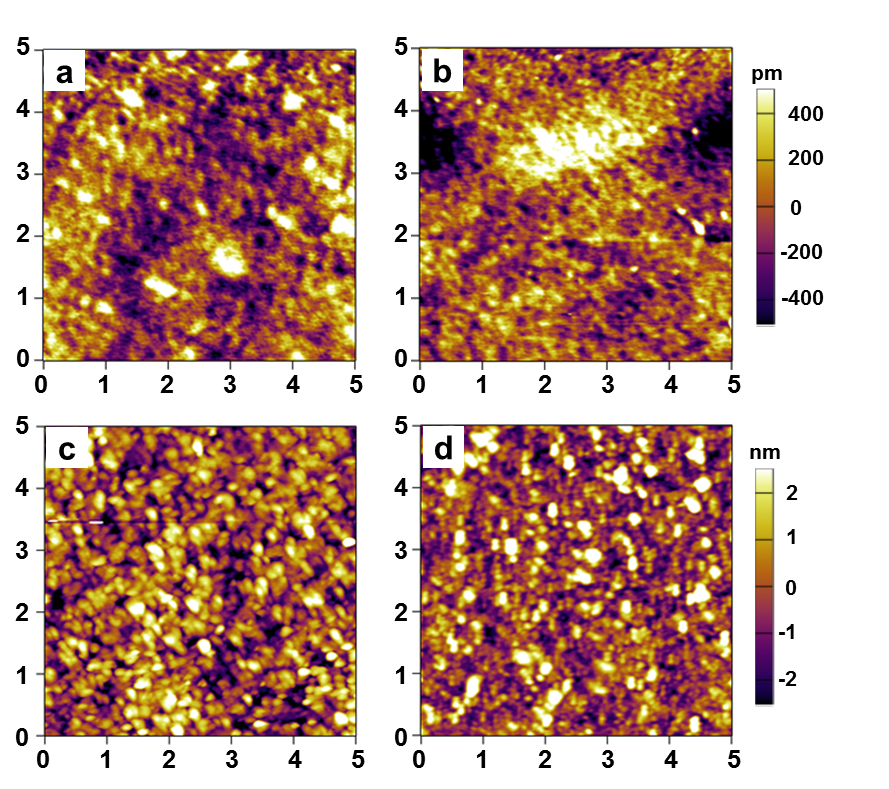
**

Figure S2 ac-AFM topographic images of glass (a), LiNbO_3_ (b), ITO-glass (c) and ITO-LiNbO_3_ (d). The images (a,b) share the color bar at pm, and the images (c,d) share the color bar at nm. The surface roughness of LiNbO_3_ is about 1 nm (a), which is similar to that of glass (b). Topographic images of ITO-glass (c) and ITO-LiNbO_3_ (d) show that they have a very similar surface morphology.

**Power conversion efficiency (*η*)**

The power conversion efficiency (*η*) is determined by the photocurrent density (*J*_sc_), the open-circuit voltage (*V*_oc_), the fill factor (*FF*) of the cell, and the incident power density (*P*_in_), according to the following formula:

$\eta=\frac{J_{sc}V_{oc}FF}{P_{in}} \times100\%$ (1)

where *FF* is defined as the ratio of the maximum power (*J*_max_*V*_max_) to the production of *J*_sc_ and *V*_oc_, i.e., *FF* = *J*_max_*V*_max_/*J*_sc_*V*_oc_. The *J*_sc_ largely depends on the photo-generated electrons and the interfacial electron recombination, while the *V*_oc_ is determined by the difference between the redox potential of the electrolyte and the Fermi level of the semiconductor.
